# Ascofuranone antibiotic is a promising trypanocidal drug for nagana

**DOI:** 10.4102/ojvr.v91i1.2115

**Published:** 2024-02-08

**Authors:** Keisuke Suganuma, Kennedy M. Mochabo, Judith K. Chemuliti, Kita Kiyoshi, Inoue Noboru, Shin-ichiro Kawazu

**Affiliations:** 1 National Research Center for Protozoan Diseases, Obihiro University of Agriculture and Veterinary Medicine, Obihiro, Japan; 2 Department of Veterinary Public Health, Pharmacology (VPHPT), Faculty of Veterinary Medicine and Surgery, Egerton University, Nakuru, Kenya; 3 Biotechnology Research Institute (BIORI), Kenya Agricultural Research Organization (KALRO), Nairobi, Kenya; 4 Department of Host-Defense Biochemistry, Institute of Tropical Medicine (NEKKEN), Nagasaki University, Nagasaki, Japan

**Keywords:** ascofuranone, trypanocide, *Trypanosoma congolense*, *Trypanosoma vivax*, antibiotic

## Abstract

Trypanosomosis is a disease complex which affects both humans and animals in sub-Saharan Africa, transmitted by the tsetse fly and distributed within the tsetse belt of Africa. But some trypanosome species, for example, *Trypanosoma brucei evansi, T. vivax, T. theileri* and *T. b. equiperdum* are endemic outside the tsetse belt of Africa transmitted by biting flies, for example, Tabanus and Stomoxys, or venereal transmission, respectively. Trypanocidal drugs remain the principal method of animal trypanosomosis control in most African countries. However, there is a growing concern that their effectiveness may be severely curtailed by widespread drug resistance. A minimum number of six male cattle calves were recruited for the study. They were randomly grouped into two (*T. vivax* and *T. congolense* groups) of three calves each. One calf per group served as a control while two calves were treatment group. They were inoculated with 10^5^ cells/mL parasites in phosphate buffered solution (PBS) in 2 mL. When parasitaemia reached 1 × 10^7.8^ cells/mL trypanosomes per mL in calves, treatment was instituted with 20 mL (25 mg/kg in 100 kg calf) ascofuranone (AF) for treatment calves, while the control ones were administered a placebo (20 mL PBS) intramuscularly. This study revealed that *T. vivax* was successfully cleared by AF but the *T. congolense* group was not cleared effectively.

**Contribution:**

There is an urgent need to develop new drugs which this study sought to address. It is suggested that the AF compound can be developed further to be a sanative drug for *T. vivax* in non-tsetse infested areas like South Americas.

## Introduction

African trypanosomosis is a disease complex in humans and animals that is caused by protozoan haemoflagellates of the genus *Trypanosoma*. The disease is transmitted by tsetse flies (*Glossina* spp.) that are found within the tsetse belt of Africa. But some trypanosome species such as *Trypanosoma brucei evansi, T. vivax, T. theileri* and *T. b. equiperdum* are endemic outside the tsetse belts, where they are transmitted by biting flies, for example, *Tabanus* and *Stomoxys* (horseflies and stable flies). The disease is discontinuously spread over 10 million km^2^ and affects populations across 37 sub-Saharan countries. In general, the animal African trypanosomosis (AAT) that affects livestock is over 100 to 150-fold more prevalent than the trypanosome infections that cause human African trypanosomosis (HAT) (Jordan 1976; Simarro et al. 2011). Where animal infection occurs, there are widespread economic and social impacts in Africa, and are now increasingly becoming a problem in South America and parts of Asia also (Steverding 2008). *T. congolense, T. vivax* and *T. brucei* are the dominant species causing AAT (Afewerk et al. 2000; Mungube et al. 2012).

Since there is no vaccine and one will not be available soon because of variable surface glycoproteins (VSGs) that allow the parasite to evade immune defence system of their hosts, the only viable anti-trypanosomosis measures available are chemotherapies (Babokhov et al. 2013). Diminazene aceturate (DA) is today the most commonly used trypanocide in cattle, sheep and goats, because of its activity against both *T. congolense* and *T. vivax* in Africa, and it has relatively low toxic side effects (Giordani et al. 2016). The DA has also been effectively used against Surra in the Philippines (Reid 2002). The recommended therapeutic dose is 3.5 mg/kg for AAT caused by *T. congolense* and *T. vivax* (but 7 mg/kg against resistant isolates) administered by intramuscularly or subcutaneously (Connor 1992).

Drug resistance against animal trypanosomes has also been reported all over the world. For example, *T. vivax* strains refractory to DA were identified in South Americas, where the compound is the first line drug to treat these infections (Desquesnes, Rocque & Peregrine 1995). Diminazene aceturate treatment failure against *T. evansi* infections in horses and mules in Thailand has also been reported, following decades of use (Tuntasuvan et al. 2003), and the same was reported in Burkina Faso also (Clausen et al. 1992). *T. evansi* strains resistant to suramin and quinapyramine have been reported in China as well as in Africa (Liao & Shen 2010; Zhou et al. 2004).

**FIGURE 1:**
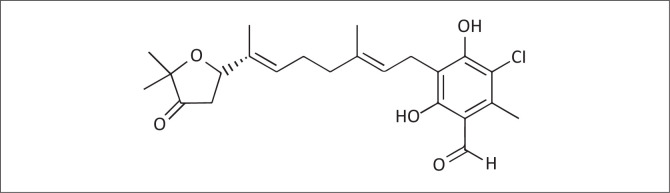
Ascofuranone structure, 2023.

Because of the challenges associated with the tsetse control, drugs have remained the most practical means of controlling the infections in livestock in many areas. No new veterinary drugs for the treatment of AAT have been released since 1985 (Anene, Onah & Nawa 2001), and there is increasing resistance to the existing trypanocides. Thus, there is an urgent need to develop new drugs. Ascofuranone (AF, Figure 1) is fungal secondary metabolite of various filamentous fungi, including *Acremonium egyptiacum* that exhibit diverse physiological activities, including antivirus, antitumour, anti-inflammatory, and hypolipidaemic activities (Araki et al. 2019; Magae et al. 1982). Most notably, AF is a strong inhibitor of cyanide-insensitive alternative oxidases (Minagawa et al. 1997; Shiba et al. 2013), and a promising drug candidate against African trypanosomosis (Nihei, Fukai & Kita 2002). In previous studies, anti-trypanosome activity *in vitro* and treatment efficacy against trypanosomosis using mice model were conducted of AF (Yabu et al. 1998, 2003, 2006). In addition, the AF was co-administered with glycerol which seemed to enhance its efficacy.

Therefore, the current study involved *in vivo* monitoring to assess the treatment efficacy of AF against *T. congolense* and *T. vivax* in experimentally infected calves.

## Materials and methods

### Study design and pre-experimental animal care

This study focussed on the efficacy of AF antibiotic using three parameters that is, parasitaemia, percent packed cell volume (%PCV), and body weights. Six 1-year old male calves (crosses of Friesian cattle breed) were internally sourced in Kenya Agricultural and Livestock Research Organization (KALRO) and recruited for the study. Their weights ranged from 80 kg to 120 kg body weight (bwt). The animals were acclimatised for 3 weeks in an animal barn house, fed on hay and supplied with clean water *ad libitum*. They were clinically examined, dewormed and vaccinated against Foot and Mouth Disease. The calves were provided with mineral salt licks and concentrates at the start of the experiment.

After acclimatisation, the calves were randomly assigned into two groups of three each. Group 1 comprised *T. congolense* group and group 2 the *T. vivax* group (hereinafter the Tc group and the Tv group, respectively). One calf in each group served as a control, while two calves were the treatment group. Each animal was ear-tagged and given a unique identification number (Tv1, Tv2, Tv3 for the Tv group, and Tc1, Tc2, Tc3 for the Tc group). The two groups were kept in separate enclosures in the barn house. The animals were screened for trypanosome using the buffy coat technique (Murray, Murray & McIntyre 1977) and pre-infection data on packed cell volume (PCV) and weight collected.

### Experimental infection and monitoring

A rodent adapted *T. vivax* isolate (KETRI 2632) and *T. congolense* KETRI 4029 (IL1180) were used in the experiment. The parasites were separately expanded in two donor Swiss white mice immuno-suppressed with cyclophosphamide at 100 mg/kg per day for three consecutive days (300 mg/kg total dose) (Wombou Toukam et al. 2011). The donor mice were examined daily for parasitaemia using the rapid matching method (Herbert & Lumsden 1976). At peak parasitaemia (1 × 10^7.8^ trypanosomes/mL for the *T. vivax* and 1 × 10^6^ trypanosomes/mL for the *T. congolense)*, the donor mice were anaesthetised using carbon dioxide, the parasites were collected through cardiac puncture in 10% ethylenediamine tetraacetic acid (EDTA) treated tubes and quantified using improved Neubauer chamber method (Petana 1963). The parasites in blood were diluted to 1.0 × 10^5^ trypanosomes/mL with Phosphate-buffered-Saline-Glucose (PSG) (44 mM NaCl, 57 mM Na_2_HPO_4_, 3 mM KH_2_PO_4_, 55 mM glucose) pH 8.0 solution. At day 0, all calves in the two groups were each inoculated into the jugular vein with 2 mL of the diluted blood containing 1.0 × 10^5^ trypanosomes/mL. The parasitaemia in both the treated (AF) and control (DA) groups were compared prospectively.

### Treatment

The Tv and Tc groups were monitored for parasitaemia three times a week by collecting and examining ear vein blood using the buffy coat technique and haematocrit centrifugation technique for PCV determination (Woo 1970). At peak parasitaemia (1 × 10^7.8^ trypanosomes per mL), calves in the treatment group, Tv1, Tv2, Tc1 and Tc2 were treated with AF at a dose rate of 25 mg/kg per day for seven days equivalent to 20 mL of AF suspension (2.5 g AF in 20 mL of tween-PBS with glycerol) per day, administered intramuscular (IM). The drug suspension was divided into two 10 mL portions and injected at two different sites to avoid any adverse reaction on the injection sites. Calves in the control group Tv3 and Tc3 were given a placebo (20 mL PBS) IM for seven consecutive days. On the last day of AF treatment, the control calf, Tv3, was treated with DA (Norbrook Kenya Ltd) at a dose rate of 3.5 mg/kg. The calves were monitored for 43 days post infection (dpi) for the Tv group and 46 dpi for the Tc group. At the end of observation period, 0.2 mL of blood from the jugular vein of each of the six calves was sub-inoculated into Swiss white mice (five mice per calf) for further parasitological monitoring for 30 days. At the end of period, the mice were sacrificed and a post-mortem examination of spleen was done.

### Data management and statistical analysis

Data were recorded in a laboratory notebook and later entered in MS Excel. They were analysed in R software version 4.3.2 (Core Team, 2023) using Kruskal-Wallis sum rank test (Chi-square) between the treatment group (Tv1, Tv2, Tc1, Tc2) and control calves (Tv3 and Tc3, respectively).

### Ethical considerations

The study was approved by KALRO Biotechnology Research Institute (KALRO-BioRI) Institutional Animal Care and Use Committee (IACUC) No. C/BioRI/4/325/II/56.

## Results

### *T. vivax* group

**FIGURE 2:**
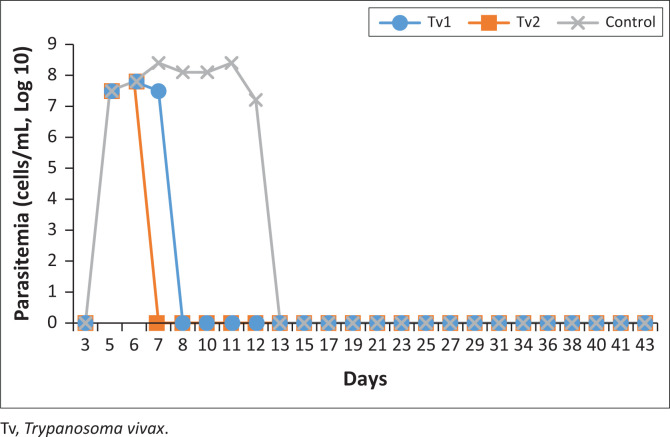
Parasitaemia of *T. vivax* infection trends. Ascofuranone treatment, day 6th to 12th in calves Tv1 and Tv2. Day 12, diminazene aceturate treatment in control calf Tv3, 2023.

The experiment begun on day one after intravenous (IV) inoculation of the trypanosomes in the calves. Ascofuranone was injected in the calves when parasitaemia reached 10^7.8^ cells/mL. On day 2 post treatment, one of the calves (Tv2) turned negative, while Tv1 parasitaemia went down to 10^7.5^ cells/mL. In the placebo calf, the parasitaemia went up 10^8.4^ cells/mL. All the AF treatment calves were negative on haematocrit centrifugation technique (HCT) on day 3 post treatment, while control calf parasitaemia was still high. On the seventh day of AF treatment, the control calf was administered with DA and on the eighth day post treatment, all calves returned a negative result. The calves were further observed for parasitaemia for another 30 days to check for any relapse and then again in mice for another 30 days. In this case, there was no relapse and in conclusion, the AF compound was successful in clearing the parasites in the two calves in the treatment group. In terms of parasitaemia, there was no difference between the control and AF treatment group (*p* = 0.096) during treatment period by Kruskal-Wallis rank sum test (Figure 2). At the end of the experiment, the sub-inoculated mice with blood from the three calves did not exhibit parasitaemia and were sacrificed to check for post-mortem changes of the spleen. The spleens of all the mice appeared normal.

**FIGURE 3:**
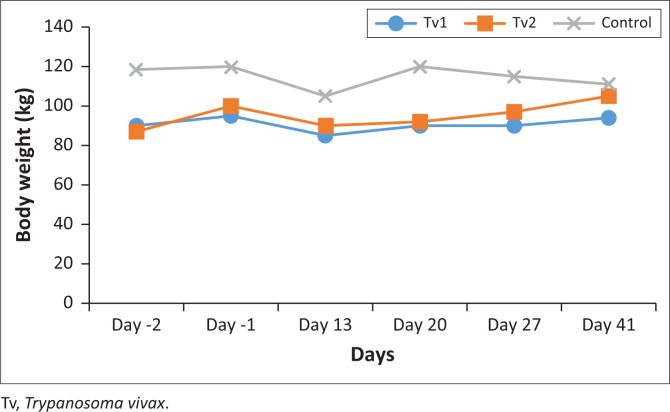
The mean weights of the Tv1 and Tv2 (treatment), and Tv3 (control), ascofuranone treatment period day 6th to 12th, 2023.

By the start of the experiment, the calves Tv1, Tv2 and Tv3 weighed 95 kg, 100 kg and 120 kg, respectively. After they were challenged and during the treatment period, the weights dropped to 85 kg, 90 kg and 105 kg, respectively. But by the end of the experiment, the calves had mean weights of 90 kg, 95 kg and 115 kg, respectively. The means were different between the treated calves and the control (*p* = 0.0026 Kruskal-Wallis rank sum test) (Figure 3).

**FIGURE 4:**
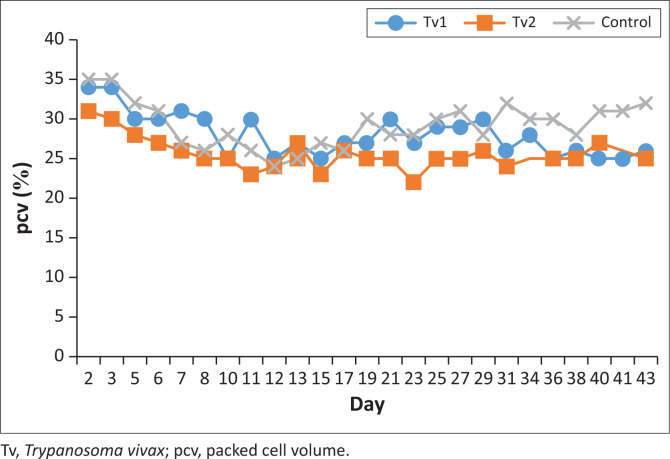
Percent packed cell volume profiles in the Tv group calves. Tv1 (Treatment), Tv2 (Treatment) and Tv3 (control). Treatment with ascofuranone started on day 6 and ended on day 12, 2023.

There were significant differences in the percentage packed cell volumes between the treatment and the control groups as shown in Figure 4 (*p* < 0.05).

### *T. congolense* group

**FIGURE 5:**
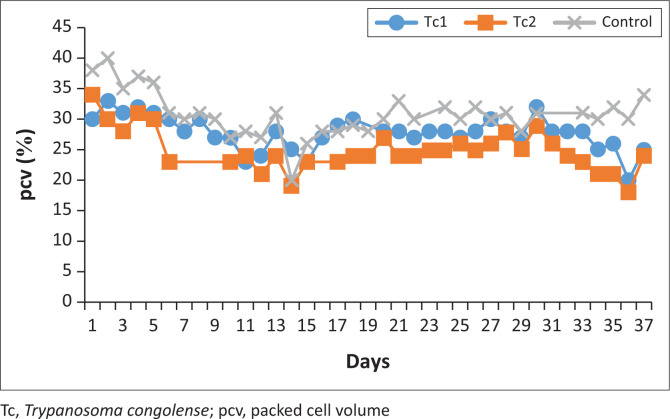
Percent packed cell volume profiles in the Tc group calves. Tc1 (Treatment), Tc2 (Treatment) and Tc3 (control). Treatment with Ascofuranone started on day 9 and ended on day 15, 2023.

The day 1 of the experiment began on the day of IV inoculation of the trypanosomes in the calves. Ascofuranone compound was injected into the calves when parasitaemia reached 10^7.8^ cells/mL on HCT, but 10^7.2^ cells/mL on a wet smear. Parasitaemia went down to 10^7.5^ cells/mL in the treated calves of 2 days post treatment, while in the placebo (Tc3) animal it remained at 10^7.8^ cells/mL. On the fifth day post treatment, Tc1 relapsed and all the calves were positive. On the seventh day of AF treatment, all calves were still positive but calf Tc3 was treated with DA, and on the eighth day post treatment only calf Tc1 was positive with parasites. On day 21, all calves returned a negative test for parasites and %PCVs improved to be normal (PCV > 25). On day 40 of the monitoring, the two AF calves in the treatment group (Tc1 and Tc2) relapsed and the subsequent 3 days their %PCV appeared to be deteriorating more especially Tc2 where it reached 21%. Because we were approaching the last day of observation but on day 43, Tc2 PCV was recorded 18% and we decided to administer DA at a dose 3.5 mg/bwt – 7 mg/bwt. There were significant differences on the %PCV between the AF treated calves and the control (*p* < 0.05) (Figure 5).

**FIGURE 6:**
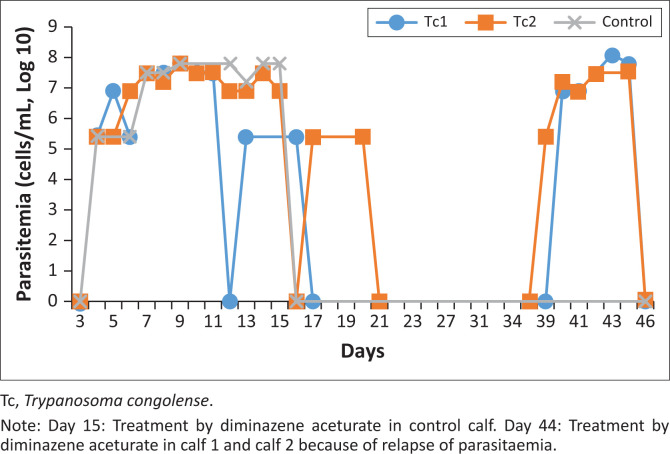
Parasitaemia *T. congolense* infection trends in Tc1 and Tc2 (treatment), Tc3 (control). Ascofuranone treatment, day 9 to day 15, 2023.

On the last day of observation (Day 46), all calves were negative of parasites; however, we sub-inoculated the calves’ blood into mice for further monitoring. All the mice were negative of trypanosome parasites after 30 days of monitoring in mice. Thereafter, the mice were sacrificed to check for post-mortem changes, especially, on the spleen. There were no significant differences between the AF calves and the control one (DA) (*p* = 0.29), whereby the AF failed to effectively cure the treatment calves as shown in Figure 6.

Because of the delays in the propagation of *T. congolense* parasites, the calves gained weight while in the barn with one recording a weight of almost 120 kg. By the time the calves were challenged with the parasites, Tc1 and Tc2 weighed 107 kg and 90 kg, respectively. The weights decreased marginally while awaiting the parasitemia to go high then weights increased slightly after the administration of AF. At the end of the experiment, the calves had a mean weight of 112 kg, 93 kg, and 120 kg for Tc1, Tc2 and Tc3, respectively. In terms of parasitaemia, there were clear differences between the control group and the AF treated group having experienced a relapse. In the Tc group, though all the calves were treated with DA, their blood were sub-inoculated into mice. No parasitaemia was recorded in mice and also their spleens appeared normal upon sacrificing the mice.

**FIGURE 7:**
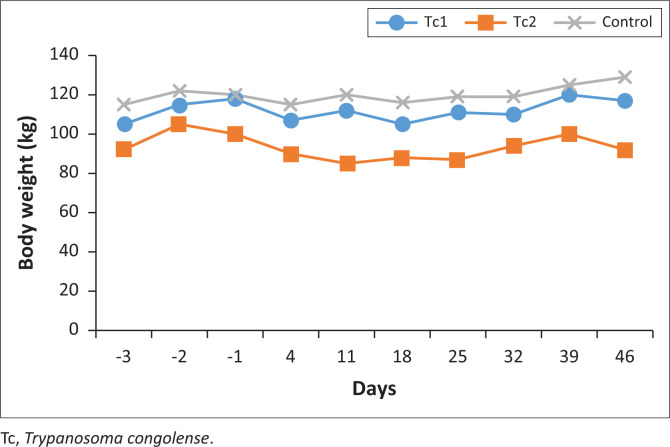
The mean weights of the Tc1 and Tc2 (treatment group), and Tc3 (control group), 2023.

Overall, the means of the treated calves and the control were different (*p* < 0.05) (Figure 7). However, it was noted that the control group had a higher initial bwt than the rest in the treatment group.

## Discussion

Most studies on *T. vivax* trypanosome species were conducted in *in vivo* laboratory models; however, very few strains of the same have been isolated that readily infect rodents, and most published *in vivo* work on this species comprises the very few mouse-infective strains, the main one being *T. vivax* Y486 and its derivatives (Gibson 2012). Against this backdrop, the isolate used in the study was rodent-adapted which would have originated from Nigeria (Yamazaki et al. 2023) and the AF effectively cured the experimentally infected calves.

Whereas the AF failed to effectively cure the Tc calves, we could however find successful results in previous studies by Yabu et al. (1998, 2003, 2006) which showed treatment efficacy of AF against *T. b. brucie* (Tbb) and Tv. Trypanosomosis caused by both species are treated by AF dose-dependently in mice. In addition, AF showed treatment efficacy against AAT caused by Tc in mice (Pers. comm.). Co-administration of glycerol could enhance the treatment efficacy because glycerol inhibits glycerol kinase activity, which is a metabolic pathway to produce ATP when AF block trypanosomal alternative oxidase. Because the sensitivity of AF is different among *Trypanosoma* species (Tv is most sensitive; Tbb moderate; Tc less sensitive), the treatment efficacy by AF against AAT model is also different among the aetiological species of trypanosomes. Based on the previous studies, the co-administration of glycerol and AF might show higher treatment efficacy. Indeed, at one point, AF appeared to have cured the Tc calves but for a relapse to occur. This merits future research to further unravel the right dosage of glycerol and AF drug co-administration to clear the parasites. Another limitation to the current study was the use a low sample size, which gave unreliable results.

*Trypanosoma congolense* is considered the most pathogenic trypanosome in cattle (followed by *T. vivax*). Apart from bovines, Tv can affect sheep, goats, horses and camels as well (Osorio et al. 2008). Now that the results for AF are promising for Tv, the drug can be further developed as an alternative because of increasing antimicrobial resistance. For all the calves whose blood were sub-inoculated into mice, no parasitaemia was recorded. At the end of the experiment, the mice were sacrificed and their spleens were checked for any enlargement. Because all spleens appeared normal, it was a further indication that there was no drug resistance against DA serendipitously. As demonstrated in our study, it was able to clear parasites within 24 h. Parasitaemia measurements were the most critical in the current study albeit monitoring the other two parameters (%PCV and weights). Because the experimental observation period was short, there were no major variations in weights and to some extent on %PCVs. However, while measuring the %PCV, it important to note that when the Tc calves at some point became anaemic and thus needed treatment intervention with DA.

To the best of our knowledge, this is the first report on AF *in vivo* studies in cattle. Taken altogether, this study revealed that the two experiments were conducted successfully in parallel, but the Tc group would not be cleared by the AF compound effectively. It is suggested that the compound can be developed further to be a sanative drug for Tv in non-tsetse infested areas like South Americas. In addition, future research is needed by testing the AF antibiotic on *T. evansi*, and check its efficacy which would as well, augment why the need to develop the compound for the aforementioned areas. Again, if feasible in the lab, the AF can be manipulated biosynthetically to come up with a compound that is effective across the *Trypanosoma* species.
