# Erratum: Ascofuranone antibiotic is a promising trypanocidal drug for nagana

**DOI:** 10.4102/ojvr.v91i1.2174

**Published:** 2024-03-22

**Authors:** Keisuke Suganuma, Kennedy M. Mochabo, Judith K. Chemuliti, Kiyoshi Kita, Noboru Inoue, Shin-ichiro Kawazu

**Affiliations:** 1 National Research Center for Protozoan Diseases, Obihiro University of Agriculture and Veterinary Medicine, Obihiro, Japan; 2 Department of Veterinary Public Health, Pharmacology (VPHPT), Faculty of Veterinary Medicine and Surgery, Egerton University, Nakuru, Kenya; 3 Biotechnology Research Institute (BIORI), Kenya Agricultural Research Organization (KALRO), Nairobi, Kenya; 4 Department of Host-Defense Biochemistry, Institute of Tropical Medicine (NEKKEN), Nagasaki University, Nagasaki, Japan

In the original article published, Suganuma, K., Mochabo, K.M., Chemuliti, J.K., Kiyoshi, K., Noboru, I. & Kawazu, S.-I., 2024, ‘Ascofuranone antibiotic is a promising trypanocidal drug for nagana’, *Onderstepoort Journal of Veterinary Research* 91(1), a2115. https://doi.org/10.4102/ojvr.v91i1.2115, two authors’ names were transposed.

Instead of:

Kita Kiyoshi and Inoue Noboru

It should be:

Kiyoshi Kita and Noboru Inoue

The publisher apologises for this error. The correction does not change the study’s findings of significance or overall interpretation of the study’s results or the scientific conclusions of the article in any way.

